# Short-Term Storage of Rooster Ejaculates: Sperm Quality and Bacterial Profile Differences in Selected Commercial Extenders

**DOI:** 10.3390/antibiotics12081284

**Published:** 2023-08-03

**Authors:** Eva Tvrdá, Michaela Petrovičová, Michal Ďuračka, Filip Benko, Tomáš Slanina, Lucia Galovičová, Miroslava Kačániová

**Affiliations:** 1 Institute of Biotechnology, Faculty of Biotechnology and Food Sciences, Slovak University of Agriculture in Nitra, Tr. A. Hlinku 2, 94976 Nitra, Slovakia; 2 Department of Neuroscience, Second Faculty of Medicine (2. LF UK), Charles University, V Úvalu 84, 15006 Prague, Czech Republic; michaela.petrovicova@lfmotol.cuni.cz; 3 AgroBioTech Research Centre, Slovak University of Agriculture in Nitra, Tr. A. Hlinku 2, 94976 Nitra, Slovakia; michal.duracka@uniag.sk; 4 Institute of Applied Biology, Faculty of Biotechnology and Food Sciences, Slovak University of Agriculture in Nitra, Tr. A. Hlinku 2, 94976 Nitra, Slovakia; xbenkof@uniag.sk (F.B.); tomas.slanina@uniag.sk (T.S.); 5 Institute of Fruit Science, Viticulture and Enology, Faculty of Horticulture and Landscape Engineering, Slovak University of Agriculture, Tr. A. Hlinku 2, 94976 Nitra, Slovakia; xgalovicoval@uniag.sk (L.G.); miroslava.kacaniova@uniag.sk (M.K.); 6 Department of Bioenergetics, Food Analysis and Microbiology, Institute of Food Technology and Nutrition, University of Rzeszow, Cwiklinskiej 1, 35-601 Rzeszow, Poland

**Keywords:** bacterial contamination, roosters, semen extender, kanamycin, MALDI-TOF, bacterial resistance

## Abstract

Bacterial contamination of semen has become an important contributor to the reduced shelf life of insemination doses in the poultry industry, which is why antibiotics (ATBs) are an important component of semen extenders. Due to a global rise in antimicrobial resistance, the aim of this study was to assess the efficiency of selected commercially available semen extenders to prevent possible bacterial contamination of rooster ejaculates. Two selected extenders free from or containing 31.2 µg/mL kanamycin (KAN) were used to process semen samples from 63 healthy Lohmann Brown roosters. Phosphate-buffered saline without ATBs was used as a control. The extended samples were stored at 4 °C for 24 h. Sperm motility, viability, mitochondrial activity, DNA integrity and the oxidative profile of each extended sample were assessed following 2 h and 24 h of storage. Furthermore, selective media were used to quantify the bacterial load and specific bacterial species were identified with matrix-assisted laser desorption/ionization time-of-flight (MALDI-TOF) mass spectrometry. The results indicate that semen extenders enriched with KAN ensured a significantly higher preservation of sperm quality in comparison to their KAN-free counterparts. Bacterial load was significantly decreased in diluents supplemented with ATBs (*p* ≤ 0.001); however, KAN alone was not effective enough to eradicate all bacteria since several *Escherichia coli*, *Enterococcus faecalis*, *Enterococcus faecium* and *Micrococcus luteus* were retrieved from samples extended in KAN-supplemented commercial extenders. As such, we may suggest that more focus should be devoted to the selection of an optimal combination and dose of antibiotics for poultry extenders, which should be accompanied by a more frequent bacteriological screening of native as well as extended poultry semen.

## 1. Introduction

A rapid global rise in primary poultry production reflects an increased consumer preference for these high-quality and relatively cheap products. In fact, over the past decades, poultry has become the most-consumed livestock commodity in the world, particularly in developing markets [1,2]. In the meantime, this commercial demand puts more pressure on us to increase the efficiency of poultry production, which by and large depends on the intensity of animal breeding and reproduction [1].

Since the 1950s, the poultry industry has witnessed remarkable progress thanks to the implementation of modern reproductive technologies. Above all, artificial insemination (AI) has become a critical pillar of intensive poultry production, as hens are being fertilized almost exclusively artificially in leading poultry-producing countries such as Israel, the USA, Brazil, France, China, or India [3]. In comparison to natural mating, AI enables a more rapid dissemination of genetic material from superior males with desirable traits to a large number of females within a short period of time while reducing the risks for disease transmission among the animals [4,5]. At the same time, AI is the only option to achieve high numbers of fertilized eggs destined for hatching in heavy, broad-breasted strains, which are characterized by unsuccessful natural mating and consequently low reproductive performance [6]. Nevertheless, a successful application of this technique relies on a good quality of semen that is inseminated close to the sperm storage tubules in the female in order to reach optimal fertility in chickens [4].

As opposed to mammalian spermatozoa, poultry sperm quickly lose their viability and function following collection, which is why semen dilution has become a popular option in the poultry industry [7]. High costs of semen processing and freezing coupled with an inherent sensitivity of poultry spermatozoa to sub-zero temperatures render cryopreservation impractical for a large-scale insemination process; thus, the most frequent strategy for poultry enterprises represents AI taking advantage of diluted semen stored either at room temperature for several hours or chilled and stored at 4–5 °C for 24–48 h [8].

Under ideal circumstances, semen extenders provide protection to male gametes and preserve their motion and fertilizing ability over time, primarily by stabilizing the plasma membrane, providing sources of energy, and preventing any harmful effects of pH and osmolarity fluctuations or oxidative stress on spermatozoa [9]. On the other hand, sperm preservation media may act as a rich reservoir of nutrients for bacterial growth if a contaminated ejaculate is diluted [10]. This is a cause for concern, particularly in the case of poultry, where the reproductive, urinary, and digestive tract share one posterior orifice (cloaca); hence, bacteriospermia is a commonly observed phenomenon [11,12,13,14,15]. Previous studies have additionally revealed that bacterial contamination of extended semen has led to sperm agglutination [16], motility inhibition [17,18] and alterations to the sperm morphology [18], rendering the affected semen sample ineffective in accomplishing fertilization. Bacterial activity may contribute to increased reactive oxygen species (ROS) levels [19], which, together with metabolic by-products and toxins, significantly contribute to a reduced shelf-life of insemination doses [10,20]. The use of contaminated semen may subsequently cause infection and infertility in the female following insemination or result in the spread of diseases in the flock [21]. Pathogenic bacteria may then be transmitted to poultry products, posing a health concern to the consumer [22].

While “growth-promoting” antibiotics in feed were abolished in 2006 and their use is only allowed to treat an ongoing infection of the flock [23], regulations governing the production of insemination doses specify that antibiotic supplements must be added to extended semen to avoid complications arising from the use of contaminated ejaculates in poultry production [10]. This practice, however, promotes the occurrence, spread and persistence of multidrug-resistant bacteria [24]. In fact, previous reports have frequently unfolded resistance among bacterial isolates from poultry semen against antibiotics commonly used as supplements in commercial semen diluents, such as penicillin, ampicillin, chloramphenicol, and tetracycline [11,12,13,14].

Hence, this study was focused on: (a) studying the bacterial profiles of extended rooster semen using matrix-assisted laser desorption/ionization time-of-flight (MALDI-TOF) mass spectrometry; (b) investigating the susceptibility of persistent bacterial isolates to antibiotics; and (c) comparing changes in the bacterial quantity and diversity of rooster ejaculates diluted with two selected commercially available poultry semen extenders with a special emphasis on their ability to preserve selected markers of sperm structural integrity and functional activity.

## 2. Results

Sixty-three Lohmann Brown rooster semen samples were collected for the experiments. Mean values for the qualitative sperm parameters and basic bacterial profile of neat ejaculates prior to dilution and storage are displayed in Table 1.

All neat semen samples tested positive for at least one bacterium. Using MALDI-TOF mass spectrometry, thirteen genera and seventeen bacterial species were identified in neat rooster semen, out of which eight species were Gram-negative and seven were classified as Gram-positive (Table 1). The predominant species included *Escherichia coli,* which was found in 82.54% of all samples; *Enterococcus faecalis,* with a 71.43% sample positivity; and *Citrobacter braakii,* which was present in 39.69% of all ejaculates.

All samples were subsequently diluted in PBS (control), poultry medium (PM) or extendil medium (EM) for poultry semen in the presence or absence of kanamycin (KAN) and stored at 4 °C for 24 h. Specific sperm quality as well as bacteriological analyses were performed at 2 h and 24 h of storage.

Data collected following 2 h of semen storage revealed that the lowest sperm motility was present in the control, representing rooster ejaculates diluted in PBS (Table 2). Significantly higher motility rates were detected in the PM as well as the EM medium in comparison with the control (*p* ≤ 0.01 with respect to AM, *p* ≤ 0.001 in the case of EM) even without the presence of KAN. Supplementation of the antibiotic led to further stabilization of the motion behavior in all diluents (*p* ≤ 0.05 in the case of PBS, *p* ≤ 0.001 with respect to PM and EM).

Semen dilution in both extenders led to a significantly higher stabilization of the sperm membrane when compared to the control (*p* ≤ 0.01; Table 2). Nevertheless, KAN supplementation caused no positive or negative effect on the integrity of the sperm membrane in either group. A similar trend was observed in the case of acrosome integrity, where a significantly higher percentage of sperm with an intact acrosome was present, particularly in the case of the EM extender in comparison with the control (*p* ≤ 0.05; Table 2). Similarly, the presence of KAN had no effect on the resulting acrosomal stability after 2 h.

Both extenders revealed significant mitochondria-stabilizing effects since a significantly higher MMP was recorded in the PM (*p* ≤ 0.05; Table 2) as well as in the EM group (*p* ≤ 0.001) in comparison with the control (Table 2). While KAN supplementation to both extenders further improved the mitochondrial activity in comparison with the control (*p* ≤ 0.01 in the case of PM, *p* ≤ 0.001 with respect to EM), no significant differences were observed among the respective counterparts.

In the case of sperm DNA integrity, semen dilution, particularly in the EM commercial extender, led to a significantly lower occurrence of cells with fragmented DNA in comparison with the control (*p* ≤ 0.05, Table 2). What is more, sperm DNA damage was even lessened in both semen extenders containing KAN when compared to antibiotic-free diluents (*p* ≤ 0.05).

The luminometric analysis revealed that both semen extenders acted as antioxidant buffers since significantly lower ROS levels were observed in both the PM (*p* ≤ 0.01) and EM (*p* ≤ 0.001) groups even without the presence of KAN when compared to the PBS control (Table 2). KAN administration fortified the ability of particularly the EM extender to prevent excessive ROS accumulation, as evidenced by significantly different ROS levels among the antibiotic-free EM group and the EM + KAN group (*p* ≤ 0.01).

A notable rise in the bacterial load was visible in all KAN-untreated groups following 2 h of storage time, although no significant differences were observed among these (Table 3). Nevertheless, KAN administration led to a significant reduction of the bacterial colonies in all groups when compared to their untreated counterparts.

Differences were also observed in the case of the bacterial profiles (Table 3). While all bacterial species identified in the neat samples withstood the dilution process and storage in the antibiotic-free groups, only *E. coli*, *E. faecalis*, *C. braakii*, *E. faecium*, *M. luteus*, *S. epidermidis* and *P. aeruginosa* were detected in the control treated with KAN. While the EM + KAN group tested positive for *E. coli*, *E. faecalis*, *E. faecium*, *M. luteus* and *S. epidermidis*, only *E. coli*, *M. luteus* and *E. faecium* were found in the PM + KAN group.

Following 24 h of storage time, both commercial extenders were able to maintain sperm motility at significantly higher rates in comparison with the control (*p* ≤ 0.05 in the case of PM, *p* ≤ 0.001 with respect to EM; Table 4). Furthermore, KAN supplementation contributed to motility stabilization as observed by significantly higher motion rates in all KAN-supplemented diluents in comparison to their antibiotic-free counterparts (*p* ≤ 0.05 in the case of PBS vs. PBS + KAN; *p* ≤ 0.05 with regards to PM vs. PM + KAN; *p* ≤ 0.05 in the case of EM vs. EM + KAN).

At the same time, significantly higher sperm viability was observed in both antibiotic-free and KAN-administered extenders when compared to the control (*p* ≤ 0.001; Table 4). KAN had no positive or negative impact on the sperm membrane’s integrity. In the case of the acrosome integrity, significant preservation of the acrosomal cap was observed, particularly in the case of the EM extender, both KAN-unsupplemented (*p* ≤ 0.05; Table 4) and KAN-supplemented variant (*p* ≤ 0.01) in comparison with the control.

The JC-1 assay at 24 h revealed significantly higher mitochondrial activity, particularly in the EM extender, when compared to the control (*p* ≤ 0.01; Table 4). Moreover, KAN offered a higher degree of protection to the mitochondrial structures in both commercial extenders in comparison to their antibiotic-free counterparts (*p* ≤ 0.05 with respect to the PM extender; *p* ≤ 0.001 in the case of the EM extender).

In the case of sperm DNA integrity, semen storage, particularly in both KAN-free extenders, led to a significantly lower occurrence of spermatozoa with damaged DNA in comparison with the control (*p* ≤ 0.01 in the case of the PM extender; *p* ≤ 0.001 with regard to the EM extender; Table 4). At the same time, sperm DNA fragmentation was further decreased in both semen extenders containing KAN when compared to their antibiotic-free versions (*p* ≤ 0.05).

Both extenders exhibited a notable antioxidant capacity even after 24 h, evidenced by a significantly lower ROS production when compared to the PBS control (*p* ≤ 0.01 with respect to the PM group; *p* ≤ 0.001 in the case of the EM group; Table 4). KAN supplementation contributed to further prevention of ROS overproduction, particularly in the case of the EM extender, as revealed by significantly decreased ROS levels in comparison to both the control (*p* ≤ 0.001) and the antibiotic-free EM group (*p* ≤ 0.05).

A high bacterial load was recorded in all KAN-untreated groups following 24 h of storage time, although without significant differences (Table 5). Inversely, KAN administration led to a significant decrease in the bacterial quantity in all enriched diluents when compared to their untreated counterparts.

With respect to changes in the bacterial profiles, all previously identified species were found in the antibiotic-free groups, while *E. coli*, *E. faecalis*, *C. braakii*, *E. faecium*, *S. epidermidis* and *P. aeruginosa* were detected in the control supplemented with KAN. *E. faecalis*, *E. faecium* and *M. luteus* were found in the EM + KAN group following 24 h, while several *E. coli* and *E. faecium* colonies were detected in the AM + KAN group.

All bacterial isolates retrieved from extended rooster semen stored for 24 h were tested for antimicrobial resistance (Table 6) against ampicillin, chloramphenicol, gentamycin, imipenem, levofloxacin, tetracycline, tigecycline and tobramycin. Inhibition zones resulting from the disc diffusion method were evaluated using Breakpoint tables for the interpretation of MICs (minimum inhibitory concentration) and zone diameters (version 13.0, valid from January 1, 2023) issued by the European Committee on Antimicrobial Susceptibility Testing (EUCAST).

All tested specimens were sensitive to gentamycin except for 11 *E. coli*, 12 *S. liquefaciens* and 2 *S. aureus* isolates. Resistance to ampicillin was observed in 17.1% of *E. coli*, 40% of *E. faecalis*, 48.2% of *C. braakii*, 33.3% of *E. faecium* and 33.4% of *S. liquefaciens* isolates. At the same time, 52.6% of *S. epidermidis* and 22.3% of *S. aureus* isolates were resistant to chloramphenicol. Around 31.9% of *E. faecalis* isolates exhibited resistance to tigecycline, while 11.1% of *E. coli*, 24.1% of *C. braaki*, 26.3% of *S. epidermidis* and 33.3% of *S. aureus* isolates were resistant to tobramycin. Only one *E. avium* was resistant to levofloxacin.

## 3. Discussion

Numerous factors are nowadays known to affect the quality of poultry ejaculates destined for AI, among which bacterial contamination has recently gained notoriety. In order to minimize the loss of sperm architecture and vitality while at the same time preventing the spread of bacterial infection within and outside the flock, readily available information concerning the bacterial profiles of native or diluted semen may be valuable for more effective handling of ejaculates and their use in the breeding process [12,14,15,18].

Our results indicate that all rooster ejaculates in this study were contaminated by different bacterial species, some of which are acknowledged as pathogens of the urinary or gastrointestinal tract, such as *E. coli*, *E. faecalis*, *K. pneumoniae* or *C. braakii*. This is in agreement with previous studies on roosters [12,14,15] or turkeys [25,26] and fortifies the hypothesis that inherent avian anatomical features predispose poultry semen to contain a considerable amount of bacterial pathogens. Most of the semen samples tested positive for *E. coli* and *E. faecalis*, which is consistent with previous reports on poultry semen [12,15,25]. Similarly to Ahmed [14] and Reiber et al. [15], we also identified *Klebsiella*, *Serratia*, *Staphylococcus* and *Micrococcus*; however, *Salmonella* or *Kluyvera* were not identified in our samples. On the other hand, we isolated *Acinetobacter*, *Proteus* and *Macrococcus*, while, interestingly, *Corynebacterium* was found in approximately one-third of ejaculates, agreeing with Alkali et al. [27], who reported that this bacterium was persistently found in extended turkey semen with a resistance to a multitude of antibiotics such as ampicillin, amoxicillin, or streptomycin.

Bacterial contamination of ejaculates may be caused by an infection or may arise during ejaculation when semen is mixed with the bacteria that colonize the reproductive tract and cloaca. At the same time, semen may be contaminated during collection and processing by unsanitary equipment, inappropriate semen conditioning or low hygiene standards [6,15]. Since all roosters in this study were healthy and exhibited no signs of systemic or urogenital infection, we may speculate that most of the bacteria found in the samples stemmed from the cloaca. Although we did take increased sanitary precautions during semen collection, bacteria found in the ejaculates may have originated from the interior walls of the cloaca. As such, we may recommend flushing the cloacal orifice shortly before collection to at least partially eliminate bacteria that may inhabit this copulatory organ. Moreover, while bacteriospermia may not be of concern under in vivo mating due to a relatively short interplay between bacteria and spermatozoa during ejaculation, extended exposure of male gametes to bacteria during semen storage may endanger the shelf life of insemination doses [18].

Bacterial presence in animal semen has forged the necessity of antibacterial agents capable of effective protection of male gametes against any detrimental effects of bacterial contamination during liquid storage. Extenders for poultry semen are available with or without antibiotics, and if antibiotic-free media are used, antibacterial substances should be administered to accommodate the European Directive 90/429/EEC [10,28]. In this study, we purchased commercially available avian media without antibiotics and chose to supplement these with 31.2 µg/mL KAN based on our previous reports [19,29]. Nevertheless, this study reveals that bacterial presence in extended semen is a common phenomenon, and antibiotic supplementation may exhibit only a limited control of bacterial persistence and growth during storage.

Our collected data indicate that while KAN was effective in eradicating most bacteria, several *E. coli*, *E. faecalis*, *C. braakii*, *E. faecium*, *S. epidermidis* and *P. aeruginosa* remained present in extended semen even after 24 h of storage. Interestingly, higher bacterial load and diversity were found in the group extended to the simplest diluent comprised of PBS and KAN, with limited availability of nutrients for bacterial growth. As such, we may speculate that the undisclosed formula of the tested extenders contains salts, sugars and other molecules known to act as antioxidants and membrane protectors that may fortify the action of the antibiotic.

Being an aminoglycoside antibiotic, KAN has a broad spectrum of activity against both Gram-positive and Gram-negative bacteria, which is why it is, along with gentamicin, a popular antibiotic supplement to semen diluents in farm animals [19,27,28,29,30]. Nonetheless, an alarming occurrence of bacterial tolerance and/or resistance towards conventional antibiotics urges us to approach antibiotic supplementation in animal production with caution. It has also been reported that up to 50% of *E. coli* isolates collected from marine birds were resistant to 14 different antibiotics or their combinations, including (30 µg) kanamycin [31]. In a different report, aminoglycoside antibiotics presented a variable degree of efficiency against *E. coli* retrieved from the semen of infertile subjects [32]. In bulls, 22% of all bacteria isolated from the semen, including *Escherichia* spp. and *Pseudomonas* spp., presented resistance to all tested antibiotics [33]. Similar observations were also reported in the case of ejaculates obtained from rams [34] and boars [35]. It has also been previously reported that enterococci and bacilli may present with a notable resistance against specifically aminoglycosides and cephalosporins [28,36,37], while *Corynebacterium* found in avian semen was studied for phenotypes of multidrug resistance [38]. Meanwhile, Alkali et al. [27] have unraveled that *E. coli* isolates retrieved from extended turkey semen were resistant to cotrimoxazole and ofloxacin, while *E. faecalis* presented with a multi-resistance to ampicillin–cloxacillin, cefuroxime, amoxicillin, ceftriaxone, streptomycin, and erythromycin. In a recent report, Rakha et al. [30] observed low sensitivity of staphylococci isolated from stored Indian red jungle fowl semen to kanamycin. These patterns of bacterial resilience towards antibiotics may at least partially provide an explanation for the persistent presence of a small group of bacteria even in diluents enriched with antibiotics. Furthermore, this collection of data strengthens the need for more regular bacteriological screening of ejaculates for AI, which may be helpful in the selection of appropriate antibiotics to extend the shelf life of stored semen and at the same time avoid complications arising from bacterial resistance.

Bacteriospermia is generally acknowledged if the bacterial count in semen exceeds 1.00 log CFU/mL [7,31,32]. This phenomenon was successfully prevented by administering KAN to both commercial semen extenders but not PBS. In theory, insemination using extended semen with a low bacterial load should not pose a threat to the female and may result in normal fertility outcomes. Furthermore, the reproductive system of hens is equipped with antibacterial proteins such as lysozyme, avian beta-defensin 11, vitelline membrane outer layer protein 1 and histone proteins H1 and H2B, which may act as a natural barrier against uropathogens that may be transmitted to the female via AI [39,40]. Nevertheless, the effects of bacterial load and female antimicrobial proteins on the insemination outcome have yet to be studied.

In this study, 24 h of semen storage resulted in reduced sperm structural integrity and functional activity, depending on the diluent, presence, or absence of the antibiotic and the corresponding extent of bacterial contamination (Supplementary Tables S2–S8). Accordingly, the diluents free from KAN were unable to control bacterial growth and activity, leading to adhesion and agglutination processes that may subsequently compromise the integrity of the sperm membranous structures [18,19,41,42] and cease the metabolic activity of male gametes [19,41]. Furthermore, bacteria such as *E. coli* or staphylococci may adhere to the flagellar structures, causing the sperm flagellum to break, knot or tear off and thus impede the normal motion behavior of the sperm cell [18,19]. The sperm’s vitality and fertilization ability may be further endangered by the release of bacterial endotoxins such as lipopolysaccharide (LPS), hemolysin, or peptidoglycan fragments, which may deteriorate the plasma membrane’s integrity, fluidity, and semi-permeability [43,44]. An increased percentage of spermatozoa with distorted membranes and/or altered acrosomes, particularly in KAN-unsupplemented samples, could be attributed to an increased bacterial load. Bacterial endotoxins are known to activate Toll-like receptors 2 and 4, as well as nuclear factor-κB, which may initiate cell death by apoptosis or necrosis [45,46,47]. On the other hand, appropriate doses of particular aminoglycosides must be carefully selected since these may cause cellular damage through ROS overgeneration, lipid peroxidation, cytochrome c release and activation of pro-apoptotic caspases [29,48,49]. Nonetheless, no significant loss of the plasma or acrosomal membrane was observed in the experimental groups supplemented with KAN, which is why we may hypothesize that the specific effects of ATB primarily depend on their concentration and time of exposure.

The release of cytotoxic molecules and pro-inflammatory cytokines as a side effect of bacterial contamination goes hand in hand with ROS overgeneration. The resulting disturbance of the oxidative milieu may represent a more serious threat to avian spermatozoa in comparison to their mammalian counterparts, which is caused by an intricate sperm cell structure in poultry [8]. Most avian spermatozoa have narrower and longer sperm heads with a smaller cell volume than mammalian gametes [50]. What is more, avian sperm membranes contain more polyunsaturated fatty acids in comparison to mammals, have a lower cholesterol/phospholipid ratio and have a lesser protein content, which renders the plasmalemma more fluid and exceptionally sensitive to lipid peroxidation and loss of function [8,12,25,51]. At the same time, bacterial contamination has been repeatedly associated with an increased rate of sperm DNA damage [12,19,25] which may be explained by two major mechanisms: (1) bacterial metabolism produces ROS, which will shift the oxidative balance towards a pro-oxidant state. Hydrogen peroxide and hydroxyl radicals will then trespass the plasma membrane and reach the nucleus, causing breakage of the sperm DNA [12,19,25,52]. (2) Bacterial endotoxins, more specifically LPS and hemolysins, either trigger caspases through ROS overproduction or cause pores in the membrane, which will lead to osmotic imbalance, mitochondrial rupture, and ROS release into the intracellular environment. Both processes will then culminate in cell death, which is accompanied by sperm DNA disintegration [43,44,53]. DNA integrity has become paramount in the assessment of male fertilization potential. It has been previously reported in men that 48% of the subjects suffering from bacteriospermia presented with a high proportion of spermatozoa with fragmented DNA [54]. What is more, the presence of potentially uropathogenic bacteria in ejaculates obtained from clinically healthy rams, bulls, turkeys, or roosters was associated with a higher degree of sperm DNA damage. Inversely, the loss of DNA integrity has become a focal side effect of ATB supplementation in sperm processing media. Recent studies have unraveled the deleterious effects of high ATB doses on the DNA stability of male gametes, particularly in the case of ciprofloxacin, doxycycline, or gentamycin [55,56]. In our case, however, a significantly decreased rate of sperm DNA damage was observed in the experimental groups supplemented with KAN in comparison to their unsupplemented counterparts. This observation supports our assumption that meticulously selected doses of KAN as an antibacterial agent may either (1) directly decrease the bacterial load and thus dimmish the chances of sperm DNA fragmentation as a result of bacteriospermia, or (2) protect sperm DNA against damage caused by the presence of bacteria and their genotoxic products. Nevertheless, KAN is not yet listed in the EUCAST Breakpoint tables, which is why further studies on its effects on prokaryotic and eukaryotic cells are needed.

On a concluding note, while MALDI-TOF mass spectrometry has emerged as a reliable and time-effective method suitable for the screening of bacteria in semen [15,25,35,36,57], its widespread use is still subject to a number of limitations, such as its inability to discriminate between related species and low analytical sensitivity without prior culture. In addition, the technique is currently unable to detect nonculturable bacteria, while the identification of slowly growing bacteria is time-consuming. As such, to obtain a complete image of the dynamic changes of the bacteriome during ex vivo processing and storage of ejaculates, molecular PCR-based diagnostics, considered the “new gold standard” in bacteriology, shall be included in future studies [20]. Finally, while ATBs are generally acknowledged as important contributors to the quality of extended semen, it is crucial to understand the impact of antibacterial compounds on sperm vitality and fertilizing ability. At the end of the day, the effects of antibacterial agents on eukaryotic cells may be multivariable, which is why their interactions with the sperm structures essential for successful fertilization need to be studied further on a more complex level.

## 4. Materials and Methods

### 4.1. Semen Collection and Dilution

Two commercial extenders, specifically the poultry medium (IMV Technologies, L’Aigle, France) and extendil medium (AMP-Lab GmbH, Münster, Germany), were purchased for the purposes of this study. Dulbecco’s phosphate-buffered saline (PBS; Sigma-Aldrich, St. Louis, MO, USA) served as a control. Each medium was used both in its antibiotic-free versions as well as variants supplemented with 31.2 µg/mL kanamycin (KAN; Sigma-Aldrich, St. Louis, MO, USA). Kanamycin was chosen based on previously gathered evidence on its biological activity and potential toxic effects on prokaryotic or eukaryotic cells, supported by previous standardization studies in our laboratory [19,28,29]. Each time, a fresh stock solution of 3.12 mg/mL KAN was prepared using PBS and subsequently added to the respective media.

Semen samples were acquired from 63 adult (35–45 weeks old) breeding Lohmann Brown roosters housed at a local broiler breeding company (Liaharenský podnik Nitra, a.s., Párovské Háje, Slovakia). Prior to each collection, the animals were observed for defecation; subsequently, their cloacae were gently washed with soap, water and dried with paper towels. Single-use gloves were changed for each rooster. Semen was collected using abdominal massage based on restraining the male and gently stroking the back from behind the wings towards the tail with firm, rapid strokes [58].

Native ejaculates were collected by a qualified technician into sterile syringes and subjected to a primary assessment of sperm volume, concentration, and motility at the collection site. Samples with a volume higher than 0.5 mL and motility above 65% were transported to the andrology laboratory in an isothermal vessel (37 °C; M&G Int, Renate, Italy) within 30 min. All ejaculates met the pre-established quality criteria and were thus included in subsequent experiments.

Each ejaculate was divided into 6 equal aliquots, and each aliquot was diluted either with the control or experimental diluent using a dilution ratio of 1:50–1:70 depending on the initial sperm concentration. Overall, 6 groups were set up for the experiments: PBS without KAN (control group); PBS supplemented with 31.2 µg/mL KAN; poultry medium (PM) without KAN; PM containing 31.2 µg/mL KAN; extendil medium (EM) without KAN; and EM administered with 31.2 µg/mL KAN. Extended semen samples were stored at 4°C. Sperm quality and bacteriological assessments were performed immediately following dilution (0 h; control group) as well as 2 h and 24 h post-dilution.

Prior to each assessment round, an aliquot of each diluted semen was pre-warmed to 37 °C and subjected to specific analysis. Furthermore, 100 µL of each sample was transferred into a sterile Eppendorf tube and stored at −20 °C for bacteriological examination [19].

### 4.2. Sperm Motility

Sperm motility (MOT; %) was assessed with the HTM TOX IVOS II. Computer-assisted sperm analysis (CASA) system (Hamilton-Thorne Biosciences, Beverly, MA, USA). For the program to differentiate between spermatozoa and bacteria, the samples were stained using the IDENT stain (Hamilton-Thorne Biosciences, Beverly, MA, USA) and analyzed under fluorescent illumination settings. Each sample was loaded into the Makler counting chamber (Sefi Medical Instruments, Haifa, Israel), and a minimum of 10 microscopic fields were automatically evaluated for motion activity [12].

### 4.3. Sperm Viability

Sperm viability expressed through plasma membrane integrity was evaluated with eosin–nigrosin vital staining. Each sample was mixed in a ratio of 1:2:2 with eosin (Eosin Y; Sigma-Aldrich, St. Louis, MO, USA) and nigrosin (Sigma-Aldrich, St. Louis, MO, USA). The mixture was smeared onto a microscopic slide and dried at laboratory temperature. All slides were assessed with the Leica DM IL LED inverted microscope (Leica Microsystems, Wetzlar, Germany) by counting 300 cells, and the proportion of membrane-intact spermatozoa is expressed in percentage (%) [12].

### 4.4. Acrosome Integrity

Acrosome integrity was evaluated with the fast green-rose Bengal staining procedure. Each sample was stained at a ratio of 1:1 with the fast green-rose Bengal staining solution (Sigma-Aldrich, St. Louis, MO, USA) and incubated at 37 °C for 70 s. Then the mixture was smeared onto a microscopic slide and air-dried. All samples were evaluated with the Leica DM IL LED inverted microscope (Leica Microsystems, Wetzlar, Germany) by counting 300 cells, and the proportion of intact acrosome spermatozoa is expressed in percentage (%) [12].

### 4.5. Mitochondrial Membrane Potential (MMP)

The mitochondrial membrane potential was evaluated with the JC-1 Mitochondrial Membrane Potential Assay kit (Cayman Chemical, Ann Arbor, MI, USA). The JC-1 dye (5.5′,6.6′-tetrachloro-1,1′,3,3′-tetraethylbenzimidazolylcarbocyanine iodide) was diluted in PBS shortly prior to analysis, and 5 μL of the JC-1 working solution were mixed with 100 μL of each sample and incubated at 37 °C for 30 min after centrifugation (2100 RPM, 25 °C, 5 min) and washed twice with a washing buffer included in the kit. All samples were then transferred to a dark 96-chamber plate and processed with the combined GloMax-Multi^+^ spectro-fluoro-luminometer (Promega, Madison, WI, USA). The resulting MMP is expressed as the ratio of JC-1 complexes to JC-1 monomers (green/red ratio) [12,19].

### 4.6. Sperm DNA Fragmentation

DNA fragmentation was quantified with the commercially available Halomax kit optimized for rooster spermatozoa (Halotech, Madrid, Spain). The cells were fixed on a microscopic slide covered by agarose. Following DNA denaturation and removal of nuclear proteins, the cells were stained with SYBR Green fluorescent stain (Sigma-Aldrich, St. Louis, MO, USA) and evaluated under the DMI6000 B fluorescent microscope (Leica Microsystems, Wetzlar, Germany). At least 300 spermatozoa were counted on each slide, and the proportion of spermatozoa with fragmented DNA is expressed in percentage (%) [29].

### 4.7. Reactive Oxygen Species (ROS) Generation

The extent of ROS production was assessed with luminol-based chemiluminescence using a 400 μL sample stained with 5 mM luminol (Sigma-Aldrich, St. Louis, MO, USA), and negative controls consisted of 400 μL of each extender. Positive controls were comprised of 400 μL of each extender, 5 mM luminol and 50 μL hydrogen peroxide (H_2_O_2_; 30%; Sigma-Aldrich, St. Louis, MO, USA). The luminescence emitted by the reaction was monitored in fifteen 1-minute cycles using the Glomax Multi^+^ combined spectro-fluoro-luminometer (Promega, Madison, WI, USA). The extent of ROS generation is expressed in relative light units (RLU)/s/10^6^ sperm [12,19].

### 4.8. Bacteriological Analysis

Plate dilution method was used for the determination of bacterial counts expressed through colony-forming units (CFUs). Basic dilution (10^−1^) was prepared by diluting 100 µL of each sample in 900 µL of distilled water and thorough mixing. Subsequent serial dilutions were prepared to reach a level of <300 CFU/mL. Diluted samples were inoculated onto blood agar base no. 2, xylose lysine deoxycholate agar, soybean casein digest agar, and Gassner agar (NutriSelect^®^ basic) (Merck, Darmstadt, Germany) and cultured under aerobic conditions (36 ± 2 °C; 24–48 h). Plates with countable colonies (30–300 CFU) were removed and counted using an automated colony counter (JP Selecta, Abrera-Barcelona, Spain) [12,19]. Ten colonies were randomly selected from each sampled plate and streaked on fresh agar plates, which were incubated at 37 °C for 24–48 h to obtain pure cultures for identification purposes [25,36,59].

### 4.9. Identification of Bacteria

Purified colonies were identified with the help of MALDI-TOF Biotyper mass spectrometry (Brucker Daltonics, Bremen, Germany). Each purified culture was mixed with 300 μL of distilled water and 900 μL of 99.8% ethanol (Centralchem, Bratislava, Slovakia) and subsequently centrifuged at 3200 RPM for 2 min. The pellet was left to dry, subsequently resuspended in 30 μL of 70% formic acid (Sigma-Aldrich, St. Louis, MO, USA), acetonitrile (Sigma-Aldrich, St. Louis, MO, USA), and centrifuged again (3500 RPM, 2 min). One microliter of the supernatant was placed on a 96-point MALDI identification plate and dried [12,19,27]. The sample was then covered with a working solution of the MALDI matrix composed of acetonitrile, ultrapure water, trifluoroacetic acid and cinnamic acid powder (Sigma-Aldrich, St. Louis, MO, USA), as previously described [27]. Bacterial identification was performed with the Microflex LT instrument and flexControl software version 3.4. Obtained spectra were compared with the MALDI Biotyper Bruker Taxonomy database (Bruker Daltonics, Bremen, Germany) [25].

### 4.10. Antibiotic Resistance Testing

Bacterial species isolated from extended boar semen were tested for antibiotic resistance. The antimicrobial susceptibility test was performed with the disc diffusion method against 10 µg of ampicillin (AMP), 10 µg of chloramphenicol (C), 10 µg of gentamycin (GEN), 10 µg of imipenem (IMP), 5 µg of levofloxacin (LEV), 30 µg of tetracycline (TET), 15 µg of tigecycline (TGC) and 10 µg of tobramycin (TOB) according to Kačániová et al. [59]. Oxoid™ antimicrobial susceptibility discs were purchased from Thermo Fisher Scientific (Waltham, MA, USA) for the purposes of the analysis. Following incubation (37 °C, 5% CO_2_, 24 h), the diameters of the inhibition zones were measured in mm and evaluated according to the EUCAST Breakpoint tables for the interpretation of MICs and zone diameters (version 13.0). Each test was repeated twice.

### 4.11. Statistical Analysis

The collected data were statistically evaluated with the GraphPad Prism program (version 8.4.3 for Mac; GraphPad Software, La Jolla, CA, USA). Descriptive statistical characteristics (mean, standard deviation) together with One-way ANOVA and the Tukey multiple comparison test were selected for the analysis. The level of significance was set at *** *p* ≤ 0.001; ** *p* ≤ 0.01; * *p* ≤ 0.05.

## 5. Conclusions

In conclusion, we may ascertain that both semen extenders selected for our experiments performed well and within current standards in terms of preserving the sperm at low temperatures. While the poultry medium seems to be more suitable for short sperm storage lasting a few hours, the extendil medium seems to be more promising in the case of prolonged avian sperm storage that may last up to 1–2 days. Nevertheless, none of the extenders was able to diminish bacterial growth without the presence of KAN, which strengthens the rationale of using antibiotic supplements as the single most important strategy to prevent excessive bacterial contamination that may endanger the shelf life of extended ejaculates. Nevertheless, a relatively high occurrence of bacterial isolates resistant to one or more antibiotics calls for appropriate strategies to avoid antibiotic overuse in poultry breeding, as well as for a more frequent bacteriological screening of native or extended ejaculates destined for artificial insemination.

## Figures and Tables

**Table 1:** Mean values for the qualitative parameters assessed in native rooster ejaculates (n = 63).

Parameter	Value (Mean ± S.D.)
Sperm motility [%]	77.16 ± 6.91
Sperm viability [%]	88.44 ± 6.39
Acrosome integrity [%]	90.41 ± 6.23
ROS production [RLU/s/10^6^ sperm]	7.62 ± 2.61
MMP [green/red ratio]	0.79 ± 0.11
Sperm DNA fragmentation [%]	19.87 ± 6.96
Bacterial load [log CFU/mL] and sample positivity	9.13 ± 3.54
	*Escherichia coli* (*E. coli*) (52/63) *Enterococcus faecalis* (*E. faecalis*) (45/63) *Citrobacter braakii* (*C. braakii*) (25/63) *Corynebacterium glutamicum* (*C. glutamicum*) (18/63) *Pseudomonas aeruginosa* (*P. aeruginosa*) (15/63) *Pseudomonas putida* (*P. putida*) (13/63) *Enterococcus faecium* (*E. faecium*) (12/63) *Micrococcus luteus* (*M. luteus*) (11/63) *Staphylococcus epidermidis* (*S. epidermidis*) (11/63) *Serratia liquefaciens* (*S. liquefaciens*) (9/63) *Streptococcus alactolyticus* (*S. alactolyticus*) (8/63) *Proteus vulgaris* (*P. vulgaris*) (7/63) *Acinetobacter baumannii* (*A. baumannii*) (6/63) *Macrococcus caseolyticus* (*M. caseolyticus*) (5/63) *Enterococcus avium* (*E. avium*) (4/63) *Klebsiella pneumoniae* (*K. pneumoniae*) (3/63) *Staphylococcus aureus* (*S. aureus*) (3/63)

ROS—reactive oxygen species, RLU—relative light units, MMP—mitochondrial membrane potential. Raw data for each sample are available in Supplementary Table S1.

**Table 2:** Qualitative and quantitative parameters of extended rooster semen in different diluents following 2 h of storage.

	Control (PBS)	PBS + KAN	PM	PM + KAN	EM	EM + KAN
Sperm motility [%]	35.10 ± 3.55	45.19 ± 2.21 ^+^	51.43 ± 4.63 **	60.00 ± 3.31 ***^,++^	58.86 ± 1.87 ***	69.33 ± 2.13 ***^,++^
Sperm viability [%]	72.40 ± 2.89	78.51 ± 2.25	82.62 ± 1.27 **	85.42 ± 1.40 **	84.17 ± 1.34 **	86.34 ± 1.93 **
Acrosome integrity [%]	79.51 ± 1.87	82.29 ± 3.03	82.97 ± 1.80	85.06 ± 3.13 *	86.55 ± 1.53 *	89.52 ± 2.62 **
MMP [red/green ratio]	0.38 ± 0.01	0.43 ± 0.02	0.45 ± 0.01 *	0.54 ± 0.01 **	0.62 ± 0.03 ***	0.68 ± 0.03 ***
Sperm DNA fragmentation [%]	32.47 ± 9.58	30.25 ± 8.52	29.04 ± 8.62	22.44 ± 7.57 **^,+^	27.45 ± 8.13 *	21.22 ± 7.66 **^,+^
ROS [RLU/s/10^6^ sperm]	17.83 ± 0.22	16.33 ± 0.10	13.07 ± 0.10 **	11.63 ± 0.04 ***	11.93 ± 0.14 ***	8.23 ± 0.15 ***^,++^
Bacterial load [log CFU/mL]	9.00 ± 0.39	3.13 ± 0.33 ***	9.63 ± 0.30	2.55 ± 0.30 ^+++^	9.88 ± 0.99	2.94 ± 0.30 ^+++^

* *p* ≤ 0.05, ** *p* ≤ 0.01, *** *p* ≤ 0.001 when compared with control (PBS); ^+^ *p* ≤ 0.05, ^++^ *p* ≤ 0.01, ^+++^ *p* ≤ 0.001 in comparison with the respective diluent without kanamycin. MMP—mitochondrial membrane potential, ROS—reactive oxygen species, RLU—relative light units, CFU—colony-forming units. PBS—phosphate-buffered saline, KAN—kanamycin, PM—poultry medium, EM—extendil medium. Time-dependent changes in the qualitative and quantitative parameters of extended rooster semen are provided in Supplementary Tables S2–S8.

**Table 3:** Bacterial profiles of extended rooster semen identified by MALDI-TOF MS Biotyper following 2 h of storage in different diluents.

	Control (PBS)	PBS + KAN	PM	PM + KAN	EM	EM + KAN
Bacterial profile and sample positivity	*E. coli* (52/63)	*E. coli* (25/63)	*E. coli* (52/63)	*E. coli* (10/63)	*E. coli* (52/63)	*E. coli* (3/63)
	*E. faecalis* (45/63)	*E. faecalis* (22/63)	*E. faecalis* (45/63)		*E. faecalis* (45/63)	*E. faecalis* (8/63)
	*C. braakii* (25/63)	*C. braakii* (9/63)	*C. braakii* (25/63)		*C. braakii* (25/63)	
	*C. glutamicum* (18/63)		*C. glutamicum* (18/63)		*C. glutamicum* (18/63)	
	*P. aeruginosa* (15/63)	*P. aeruginosa* (3/63)	*P. aeruginosa* (15/63)		*P. aeruginosa* (15/63)	
	*P. putida* (13/63)		*P. putida* (13/63)		*P. putida* (13/63)	
	*E. faecium* (12/63)	*E. faecium* (6/63)	*E. faecium* (12/63)	*E. faecium* (3/63)	*E. faecium* (12/63)	*E. faecium* (4/63)
	*M. luteus* (11/63)	*M. luteus* (7/63)	*M. luteus* (11/63)	*M. luteus* (5/63)	*M. luteus* (11/63)	*M. luteus* (6/63)
	*S. epidermidis* (11/63)	*S. epidermidis* (5/63)	*S. epidermidis* (11/63)		*S. epidermidis* (11/63)	*S. epidermidis* (3/63)
	*S. liquefaciens* (9/63)		*S. liquefaciens* (9/63)		*S. liquefaciens* (9/63)	
	*S. alactolyticus* (8/63)		*S. alactolyticus* (8/63)		*S. alactolyticus* (8/63)	
	*P. vulgaris* (7/63)		*P. vulgaris* (7/63)		*P. vulgaris* (7/63)	
	*A. baumannii* (6/63)		*A. baumannii* (6/63)		*A. baumannii* (6/63)	
	*M. caseolyticus* (5/63)		*M. caseolyticus* (5/63)		*M. caseolyticus* (5/63)	
	*E. avium* (4/63)		*E. avium* (4/63)		*E. avium* (4/63)	
	*K. pneumoniae* (3/63)		*K. pneumoniae* (3/63)		*K. pneumoniae* (3/63)	
	*S. aureus* (3/36)		*S. aureus* (3/36)		*S. aureus* (3/36)	

PBS—phosphate-buffered saline, KAN—kanamycin, PM—poultry medium, EM—extendil medium.

**Table 4:** Qualitative and quantitative parameters of extended rooster semen in different diluents following 24 h of storage.

	Control (PBS)	PBS + KAN	PM	PM + KAN	EM	EM + KAN
Sperm motility [%]	12.91 ± 2.42	17.33 ± 3.06 ^+^	21.71 ± 1.16 *	30.71 ± 0.49 **^,+^	42.29 ± 2.09 ***	50.71 ± 1.58 ***^,+^
Sperm viability [%]	50.54 ± 4.58	51.47 ± 3.28	70.30 ± 3.67 ***	72.40 ± 3.39 ***	76.59 ± 3.20 ***	78.67 ± 2.81 ***
Acrosome integrity [%]	77.27 ± 2.51	77.78 ± 2.03	79.84 ± 3.50	81.25 ± 1.64 *	81.91 ± 1.84 *	87.51 ± 1.36 **^,+^
MMP [red/green ratio]	0.17 ± 0.01	0.24 ± 0.01 ^+^	0.23 ± 0.01	0.33 ± 0.01 **^,+^	0.38 ± 0.01 **	0.53 ± 0.03 ***^,+++^
Sperm DNA fragmentation [%]	52.16 ± 7.88	49.01 ± 8.63	39.88 ± 7.18 **	30.31 ± 8.57 **^,+^	35.15 ± 9.22 ***	27.09 ± 6.29 ***^,+^
ROS [RLU/s/10^6^ sperm]	33.48 ± 0.55	29.15 ± 0.97 *	26.89 ± 0.53 *	25.15 ± 0.52 **	19.08 ± 0.21 ***	16.60 ± 0.37 ***^,+^
Bacterial load [log CFU/mL]	10.12 ± 1.13	2.67 ± 0.32 ^+++^	10.65 ± 1.40	0.97 ± 0.46 ***^,+++^	10.59 ± 2.07	0.75 ± 0.29 ***^,+++^

* *p* ≤ 0.05, ** *p* ≤ 0.01, *** *p* ≤ 0.001 when compared with control (PBS); ^+^ *p* ≤ 0.05, ^+++^ *p* ≤ 0.001 in comparison with the same diluent without kanamycin. MMP—mitochondrial membrane potential, ROS—reactive oxygen species, RLU—relative light units, CFU—colony-forming units. PBS—phosphate-buffered saline, KAN—kanamycin, PM—poultry medium, EM—extendil medium. Time-dependent changes in the qualitative and quantitative parameters of extended rooster semen are provided in Supplementary Tables S2–S8.

**Table 5:** Bacterial profiles of extended rooster semen identified by MALDI-TOF MS Biotyper following 24 h of storage in different diluents.

	Control (PBS)	PBS + KAN	PM	PM + KAN	EM	EM + KAN
Bacterial profile and sample positivity	*E. coli* (52/63)	*E. coli* (15/63)	*E. coli* (52/63)	*E. coli* (8/63)	*E. coli* (52/63)	
	*E. faecalis* (45/63)	*E. faecalis* (10/63)	*E. faecalis* (45/63)		*E. faecalis* (45/63)	*E. faecalis* (2/63)
	*C. braakii* (25/63)	*C. braakii* (8/63)	*C. braakii* (25/63)		*C. braakii* (25/63)	
	*C. glutamicum* (18/63)		*C. glutamicum* (18/63)		*C. glutamicum* (18/63)	
	*P. aeruginosa* (15/63)	*P. aeruginosa* (3/63)	*P. aeruginosa* (15/63)		*P. aeruginosa* (15/63)	
	*P. putida* (13/63)		*P. putida* (13/63)		*P. putida* (13/63)	
	*E. faecium* (12/63)	*E. faecium* (3/63)	*E. faecium* (12/63)	*E. faecium* (3/63)	*E. faecium* (12/63)	*E. faecium* (3/63)
	*M. luteus* (11/63)		*M. luteus* (11/63)		*M. luteus* (11/63)	*M. luteus* (3/63)
	*S. epidermidis* (11/63)	*S. epidermidis* (5/63)	*S. epidermidis* (11/63)		*S. epidermidis* (11/63)	
	*S. liquefaciens* (9/63)		*S. liquefaciens* (9/63)		*S. liquefaciens* (9/63)	
	*S. alactolyticus* (8/63)		*S. alactolyticus* (8/63)		*S. alactolyticus* (8/63)	
	*P. vulgaris* (7/63)		*P. vulgaris* (7/63)		*P. vulgaris* (7/63)	
	*A. baumannii* (6/63)		*A. baumannii* (6/63)		*A. baumannii* (6/63)	
	*M. caseolyticus* (5/63)		*M. caseolyticus* (5/63)		*M. caseolyticus* (5/63)	
	*E. avium* (4/63)		*E. avium* (4/63)		*E. avium* (4/63)	
	*K. pneumoniae* (3/63)		*K. pneumoniae* (3/63)		*K. pneumoniae* (3/63)	
	*S. aureus* (3/36)		*S. aureus* (3/36)		*S. aureus* (3/36)	

PBS—phosphate-buffered saline, KAN—kanamycin, PM—poultry medium, EM—extendil medium.

**Table 6:** Resistance profiles of bacterial isolates recovered from extended rooster semen following 24 h of storage.

		AMP	GEN	C	TET	IMP	TOB	TGC	LEV
*E. coli* (181 isolates)	S	27.7%	93.9%	ND	ND	88.4%	88.9%	100.0%	100.0%
	I	55.2%	0.0%			11.6%	0.0%	0.0%	0.0%
	R	17.1%	6.1%			0.0%	11.1%	0.0%	0.0%
*E. faecalis* (147 isolates)	S	31.9%	ND	ND	ND	83.6%	ND	68.1%	95.2%
	I	30.0%				11.6%		0.0%	0.0%
	R	40.0%				4.8%		31.9%	4.8%
*C. braakii* (83 isolates)	S	0.0%	100.0%	ND	ND	100.0%	50.6%	100.0%	75.9%
	I	51.8%	0.0%			0.0%	25.3%	0.0%	24.1%
	R	48.2%	0.0%			0.0%	24.1%	0.0%	0.0%
*C. glutamicum* (54 isolates)	S	ND	ND	ND	75.9%	ND	ND	ND	ND
	I				24.1%				
	R				0.0%				
*P. aeruginosa* (48 isolates)	S	ND	ND	ND	ND	62.5% 0.0% 37.5%	70.9% 29.1% 0.0%	ND	79.2% 20.8% 0.0%
	I								
	R								
*P. putida* (39 isolates)	S	ND	ND	ND	ND	74.4%	92.3%	ND	77.0%
	I					7.7%	7.7%		23.0%
	R					17.9%	0.0%		0.0%
*E. faecium* (45 isolates)	S	33.4%	ND	ND	ND	66.6%	ND	100.0%	100.0%
	I	33.3%				0.0%		0.0%	0.0%
	R	33.3%				33.4%		0.0%	0.0%
*M. luteus* (36 isolates)	S	ND	100.0%	ND	55.5%	ND	100.0%	100.0%	77.8%
	I		0.0%		44.5%		0.0%	0.0%	22.2%
	R		0.0%		0.0%		0.0%	0.0%	0.0%
*S. epidermidis* (38 isolates)	S	ND	100.0%	ND	0.0%	ND	52.6%	100.0%	100.0%
	I		0.0%		47.4%		21.1%	0.0%	0.0%
	R		0.0%		52.6%		26.3%	0.0%	0.0%
*S. liquefaciens* (27 isolates)	S	33.3%	55.6%	ND	ND	84.2%	100.0%	88.9%	100.0%
	I	33.3%	18.5%			15.8%	0.0%	0.0%	0.0%
	R	33.4%	25.9%			0.0%	0.0%	11.1%	0.0%
*S. alactolyticus* (24 isolates)	S	ND	ND	ND	100.0%	ND	ND	100.0%	100.0%
	I				0.0%			0.0%	0.0%
	R				0.0%			0.0%	0.0%
*P. vulgaris* (21 isolates)	S	66.7%	100.0%	ND	ND	100.0%	100.0%	100.0%	100.0%
	I	33.3%	0.0%			0.0%	0.0%	0.0%	0.0%
	R	0.0%	0.0%			0.0%	0.0%	0.0%	0.0%
*A. baumannii* (18 isolates)	S	ND	100.0%	ND	ND	100.0%	100.0%	ND	100.0%
	I		0.0%			0.0%	0.0%		0.0%
	R		0.0%			0.0%	0.0%		0.0%
*M. caseolyticus* (15 isolates)	S	ND	100.0%	ND	100.0%	ND	100.0%	100.0%	100.0%
	I		0.0%		0.0%		0.0%	0.0%	0.0%
	R		0.0%		0.0%		0.0%	0.0%	0.0%
*E. avium* (12 isolates)	S	83.4%	ND	ND	ND	75.0%	ND	91.6%	91.6%
	I	16.6%				25.0%		8.4%	0.0%
	R	0.0%				0.0%		0.0%	8.4%
*K. pneumoniae* (9 isolates)	S	100.0%	100.0%	ND	ND	100.0%	100.0%	100.0%	100.0%
	I	0.0%	0.0%			0.0%	0.0%	0.0%	0.0%
	R	0.0%	0.0%			0.0%	0.0%	0.0%	0.0%
*S. aureus* (9 isolates)	S	ND	77.8%	ND	33.3%	ND	44.4%	100.0%	100.0%
	I		22.2%		44.4%		22.3%	0.0%	0.0%
	R		0.0%		22.3%		33.3%	0.0%	0.0%

AMP—ampicillin, C—chloramphenicol, GEN—gentamycin, IMP—imipenem, LEV—levofloxacin, TET—tetracycline, TGC—tigecycline, TOB—tobramycin. ND—not defined, S—sensitive, I—intermediate, R—resistant.

## Data Availability

The data presented in this study are available on request from the corresponding author.

## Supplementary Materials

The following are available online at https://www.mdpi.com/article/10.3390/antibiotics12081284/s1, Supplementary Table S1: Raw data for the qualitative and quantitative parameters of native rooster ejaculates included in the experiments. Supplementary Table S2: Time-dependent changes in the sperm motility of extended rooster semen in different diluents. Supplementary Table S3: Time-dependent changes in the sperm viability of extended rooster semen in different diluents. Supplementary Table S4: Time-dependent changes in the sperm acrosome integrity of extended rooster semen in different diluents. Supplementary Table S5: Time-dependent changes in the sperm mitochondrial membrane potential in extended rooster semen in different diluents. Supplementary Table S6: Time-dependent changes in the sperm TUNEL positivity in extended rooster semen in different diluents. Supplementary Table S7: Time-dependent changes in ROS production by spermatozoa in extended rooster semen in different diluents. Supplementary Table S8: Time-dependent changes in the bacterial load of extended rooster semen in different diluents.
