# Supplementary material for: Short-Term Storage of Rooster Ejaculates: Sperm Quality and Bacterial Profile Differences in Selected Commercial Extenders
Source: Antibiotics (Basel). 2023 Aug 3;12(8):1284. doi: 10.3390/antibiotics12081284 (PMC10451222; doi:10.3390/antibiotics12081284)
Supplement: Supplementary file 1 [file antibiotics-12-01284-s001.zip › antibiotics-2489359-supplementary.pdf]

Supplementary Table S1. Raw data for the qualitative and quantitative parameters of native rooster ejaculates included in the experiments.

No.	Sperm motility [%]	Sperm viability [%]	Acrosome integrity [%]	ROS production [RLU/s/10^6 sperm]	MMP [green/red ratio]	Sperm DNA fragmentation [%]	Bacterial load [log CFU/mL]
1	77.00	82.00	84.00	7.26	0.78	19.60	10.55
2	80.00	93.00	90.00	6.88	0.81	20.20	6.74
3	66.00	80.00	81.00	10.12	0.55	30.80	15.47
4	67.00	82.00	83.00	12.12	0.57	28.40	16.23
5	74.00	85.00	90.00	8.23	0.73	29.40	11.25
6	71.00	86.00	89.00	9.05	0.70	20.40	14.41
7	70.00	87.00	84.00	9.96	0.72	25.60	10.58
8	66.00	79.00	85.00	12.97	0.63	26.20	18.42
9	69.00	79.00	82.00	10.03	0.62	28.60	17.22
10	78.00	85.00	86.00	6.55	0.74	23.60	10.12
11	75.00	79.00	80.00	7.14	0.72	25.00	9.56
12	70.00	85.00	84.00	5.52	0.76	28.00	9.87
13	68.00	76.00	78.00	11.58	0.62	26.6	12.54
14	69.00	79.00	81.00	12.13	0.59	29.400	13.02
15	71.00	85.00	88.00	8.84	0.78	25.80	9.55
16	82.00	94.00	95.00	5.23	0.90	16.80	6.84
17	67.00	79.00	78.00	10.67	0.67	28.80	15.2
18	82.00	92.00	94.00	4.45	0.91	17.20	5.48
19	81.00	92.00	95.00	5.02	0.93	16.00	6.28
20	77.00	89.00	90.00	8.78	0.77	23.60	10.03
21	72.00	88.00	87.00	9.20	0.91	31.40	10.23
22	71.00	89.00	88.00	9.22	0.89	19.80	10.00
23	73.00	87.00	89.00	6.98	0.78	27.20	10.94
24	66.00	79.00	80.00	13.25	0.59	29.00	12.45
25	68.00	76.00	81.00	11.22	0.62	29.80	13.54
26	71.00	84.00	87.00	9.56	0.7	23.40	11.47
27	76.00	86.00	88.00	8.01	0.76	27.20	9.58
28	68.00	75.00	79.00	10.68	0.68	25.60	12.24
29	68.00	79.00	83.00	11.96	0.62	26.80	12.12
30	76.00	85.00	88.00	9.45	0.81	27.20	10.02
31	82.00	92.00	94.00	4.25	0.90	13.80	6.15
32	87.00	94.00	95.00	4.06	0.94	11.00	5.57
33	67.00	86.00	87.00	11.34	0.63	25.40	13.38
34	82.00	91.00	96.00	5.21	0.86	20.40	7.02
35	78.00	90.00	94.00	7.89	0.79	25.40	9.63
36	77.00	92.00	93.00	8.56	0.78	22.60	11.85
37	74.00	83.00	89.00	9.46	0.77	26.00	10.45
38	83.00	95.00	96.00	4.99	0.86	13.60	5.26
39	81.00	95.00	96.00	5.67	0.89	14.80	6.35
40	80.00	92.00	94.00	5.24	0.85	16.20	6.59
41	79.00	87.00	90.00	8.59	0.78	18.00	7.77
42	75.00	88.00	90.00	9.88	0.75	20.60	10.02
43	74.00	86.00	89.00	8.64	0.77	16.40	11.11
44	71.00	87.00	91.00	9.52	0.71	15.00	10.19

45	84.00	93.00	94.00	5.68	0.90	15.00	4.57
46	86.00	95.00	98.00	4.97	0.92	13.60	4.15
47	87.00	97.00	97.00	5.07	0.94	12.00	5.20
48	88.00	98.00	99.00	4.26	0.92	12.20	4.72
49	81.00	93.00	94.00	5.03	0.85	13.40	5.64
50	86.00	96.00	97.00	5.22	0.91	10.40	4.94
51	82.00	95.00	98.00	5.25	0.85	16.20	4.77
52	85.00	95.00	98.00	4.99	0.84	10.60	5.06
53	86.00	97.00	99.00	5.18	0.87	10.20	5.26
54	77.00	88.00	90.00	9.33	0.77	19.20	11.16
55	79.00	87.00	90.00	8.95	0.78	20.20	9.45
56	86.00	97.00	98.00	6.24	0.91	11.00	5.44
57	87.00	96.00	97.00	5.12	0.96	10.80	5.99
58	88.00	97.00	97.00	4.63	0.94	9.80	5.28
59	80.00	96.00	98.00	5.19	0.86	12.00	6.93
60	85.00	93.00	97.00	5.18	0.87	8.80	6.55
61	83.00	94.00	98.00	5.06	0.80	10.40	5.78
62	87.00	94.00	98.00	4.89	0.90	9.60	6.19
63	85.00	97.00	98.00	4.61	0.89	9.60	5.06

MMP—mitochondrial membrane potential, ROS—reactive oxygen species, RLU – relative light units, CFU – colony-forming units.

Supplementary Table S2. Time-dependent changes in the sperm motility [%] of extended rooster semen in different diluents.

	0 h	2 h	24 h
Control (PBS)	77.16 ± 6.91	35.10 ± 3.55 ***	12.91 ± 2.42 ***
PBS + KAN		45.19 ± 2.21 ***	17.33 ± 3.06 ***
PM		51.43 ± 4.63 **	21.71 ± 1.16 ***
PM + KAN		60.00 ± 3.31 *	30.71 ± 0.49 ***
EM		58.86 ± 1.87 **	42.29 ± 2.09 ***
EM + KAN		69.33 ± 2.13	50.71 ± 1.58 **

* $p \leq 0.05$, ** $p \leq 0.01$, *** $p \leq 0.001$ when compared with control (PBS) at 0 h. PBS – phosphate buffered saline, KAN – kanamycin, PM – Poultry medium, EM – Extendil medium.

The collected data were statistically evaluated with the GraphPad Prism program (version 8.4.3 for Mac; GraphPad Software, La Jolla, CA, USA). One-way ANOVA and Dunnett's test were selected to compare the control and experimental groups at 2 h and 24 h against the control at 0h exclusively. The level of significance was set at *** $p \leq 0.001$; ** $p \leq 0.01$; * $p \leq 0.05$.

Supplementary Table S3. Time-dependent changes in the sperm viability [%] of extended rooster semen in different diluents.

	0 h	2 h	24 h
Control (PBS)	88.44 ± 6.39	72.40 ± 2.89 **	50.54 ± 4.58 ***
PBS + KAN		78.51 ± 2.25 *	51.47 ± 3.28 ***
PM		82.62 ± 1.27	70.30 ± 3.67 **
PM + KAN		85.42 ± 1.40	72.40 ± 3.39 **
EM		84.17 ± 1.34	76.59 ± 3.20 *
EM + KAN		86.34 ± 1.93	78.67 ± 2.81 *

*P≤ 0.05, **P≤ 0.01, ***P≤ 0.001 when compared with control (PBS) at 0h. PBS – phosphate buffered saline, KAN – kanamycin, PM – Poultry medium, EM – Extendil medium.

The collected data were statistically evaluated with the GraphPad Prism program (version 8.4.3 for Mac; GraphPad Software, La Jolla, CA, USA). One-way ANOVA and Dunnett's test were selected to compare the control and experimental groups at 2 h and 24 h against the control at 0h exclusively. The level of significance was set at *** $p \leq 0.001$; ** $p \leq 0.01$; * $p \leq 0.05$.

Supplementary Table S4. Time-dependent changes in the sperm acrosome integrity [%] of extended rooster semen in different diluents.

	0 h	2 h	24 h
Control (PBS)	90.41 ± 6.23	79.51 ± 1.87 **	77.27 ± 2.51 **
PBS + KAN		82.29 ± 3.03 *	77.78 ± 2.03 **
PM		82.97 ± 1.80	79.84 ± 3.50 **
PM + KAN		85.06 ± 3.13	81.25 ± 1.64 *
EM		86.55 ± 1.53	81.91 ± 1.84 *
EM + KAN		89.52 ± 2.62	87.51 ± 1.36

* $p \leq 0.05$, ** $p \leq 0.01$when compared with control (PBS) at 0 h. PBS – phosphate buffered saline, KAN – kanamycin, PM – Poultry medium, EM – Extendil medium.

The collected data were statistically evaluated with the GraphPad Prism program (version 8.4.3 for Mac; GraphPad Software, La Jolla, CA, USA). One-way ANOVA and Dunnett's test were selected to compare the control and experimental groups at 2h and 24h against the control at 0h exclusively. The level of significance was set at *** $p \leq 0.001$; ** $p \leq 0.01$; * $p \leq 0.05$.

Supplementary Table S5. Time-dependent changes in the sperm mitochondrial membrane potential [red/green ratio] in extended rooster semen in different diluents.

	0 h	2 h	24 h
Control (PBS)	0.79 ± 0.11	0.38 ± 0.01 ***	0.17 ± 0.00 ***
PBS + KAN		0.43 ± 0.02 ***	0.24 ± 0.01 ***
PM		0.45 ± 0.00 **	0.23 ± 0.01 ***
PM + KAN		0.54 ± 0.00 **	0.33 ± 0.00 ***
EM		0.62 ± 0.03 *	0.38 ± 0.00 ***
EM + KAN		0.68 ± 0.03	0.53 ± 0.03 **

* $p \leq 0.05$, ** $p \leq 0.01$, *** $p \leq 0.001$ when compared with control (PBS) at 0 h. PBS – phosphate buffered saline, KAN – kanamycin, PM – Poultry medium, EM – Extendil medium.

The collected data were statistically evaluated with the GraphPad Prism program (version 8.4.3 for Mac; GraphPad Software, La Jolla, CA, USA). One-way ANOVA and Dunnett's test were selected to compare the control and experimental groups at 2 h and 24 h against the control at 0h exclusively. The level of significance was set at *** $p \leq 0.001$; ** $p \leq 0.01$; * $p \leq 0.05$.

Supplementary Table S6. Time-dependent changes in sperm DNA fragmentation [%] in extended rooster semen in different diluents.

	0 h	2 h	24 h
Control (PBS)	19.87 ± 6.96	32.47 ± 9.58 **	52.16 ± 7.88 ***
PBS + KAN		30.25 ± 8.52 *	49.01 ± 8.63 ***
PM		29.04 ± 8.62 *	39.88 ± 7.18 ***
PM + KAN		22.44 ± 7.57	30.31 ± 8.57 **
EM		27.45 ± 8.13 *	35.15 ± 9.22 **
EM + KAN		21.22 ± 7.66	27.09 ± 6.29 *

* $p \leq 0.05$, ** $p \leq 0.01$, ***$p \leq 0.001$ when compared with control (PBS) at 0 h. PBS – phosphate buffered saline, KAN – kanamycin, PM – Poultry medium, EM – Extendil medium.

The collected data were statistically evaluated with the GraphPad Prism program (version 8.4.3 for Mac; GraphPad Software, La Jolla, CA, USA). One-way ANOVA and Dunnett's test were selected to compare the control and experimental groups at 2 h and 24 h against the control at 0h exclusively. The level of significance was set at *** $p \leq 0.001$; ** $p \leq 0.01$; * $p \leq 0.05$.

Supplementary Table S7. Time-dependent changes in ROS production by spermatozoa [RLU/s/10^6 sperm] in extended rooster semen in different diluents.

	0 h	2 h	24 h
Control (PBS)	7.62 ± 2.61	17.83 ± 0.22 *	33.48 ± 0.55 ***
PBS + KAN		16.33 ± 0.10 *	29.15 ± 0.97 ***
PM		13.07 ± 0.10 *	26.89 ± 0.53 **
PM + KAN		11.63 ± 0.04	25.15 ± 0.52 **
EM		11.93 ± 0.14	19.08 ± 0.21 **
EM + KAN		8.23 ± 0.15	16.60 ± 0.37*

* $p \leq 0.05$, ** $p \leq 0.01$, *** $p \leq 0.001$ when compared with control (PBS) at 0 h. PBS – phosphate buffered saline, KAN – kanamycin, PM – Poultry medium, EM – Extendil medium.

The collected data were statistically evaluated with the GraphPad Prism program (version 8.4.3 for Mac; GraphPad Software, La Jolla, CA, USA). One-way ANOVA and Dunnett's test were selected to compare the control and experimental groups at 2 h and 24 h against the control at 0h exclusively. The level of significance was set at *** $p \leq 0.001$; ** $p \leq 0.01$; * $p \leq 0.05$.

Supplementary Table S8. Time-dependent changes in the bacterial load [log CFU/mL] of extended rooster semen in different diluents.

	0 h	2 h	24 h
Control (PBS)	9.13 ± 3.54	9.00 ± 0.39	10.12±1.13
PBS + KAN		3.13 ± 0.33 **	2.67±0.32 **
PM		9.63 ± 0.30	10.65±1.40
PM + KAN		2.55 ± 0.30 **	0.97±0.46 ***
EM		9.88 ± 0.99	10.59±2.07
EM + KAN		2.94 ± 0.30 **	0.75±0.29 ***

** $p \leq 0.01$, *** $p \leq 0.001$ when compared with control (PBS) at 0 h. PBS – phosphate buffered saline, KAN – kanamycin, PM – Poultry medium, EM – Extendil medium.
